# Effects of Menthol Supplementation in Feedlot Cattle Diets on the Fecal Prevalence of Antimicrobial-Resistant *Escherichia coli*

**DOI:** 10.1371/journal.pone.0168983

**Published:** 2016-12-28

**Authors:** C. C. Aperce, R. Amachawadi, C. L. Van Bibber-Krueger, T. G. Nagaraja, H. M. Scott, J. Vinasco-Torre, J. S. Drouillard

**Affiliations:** 1 Departments of Animal Sciences and Industry, Kansas State University, Manhattan, Kansas, United States of America; 2 Diagnostic Medicine and Pathobiology, Kansas State University, Manhattan, Kansas, United States of America; Universidade de Aveiro, PORTUGAL

## Abstract

The pool of antimicrobial resistance determinants in the environment and in the gut flora of cattle is a serious public health concern. In addition to being a source of human exposure, these bacteria can transfer antibiotic resistance determinants to pathogenic bacteria and endanger the future of antimicrobial therapy. The occurrence of antimicrobial resistance genes on mobile genetic elements, such as plasmids, facilitates spread of resistance. Recent work has shown *in vitro* anti-plasmid activity of menthol, a plant-based compound with the potential to be used as a feed additive to beneficially alter ruminal fermentation. The present study aimed to determine if menthol supplementation in diets of feedlot cattle decreases the prevalence of multidrug-resistant bacteria in feces. Menthol was included in diets of steers at 0.3% of diet dry matter. Fecal samples were collected weekly for 4 weeks and analyzed for total coliforms counts, antimicrobial susceptibilities, and the prevalence of *tet* genes in *E*. *coli* isolates. Results revealed no effect of menthol supplementation on total coliforms counts or prevalence of *E*. *coli* resistant to amoxicillin, ampicillin, azithromycin, cefoxitin, ceftiofur, ceftriaxone, chloramphenicol, ciprofloxacin, gentamicin, kanamycin, nalidixic acid, streptomycin, sulfisoxazole, and sulfamethoxazole; however, 30 days of menthol addition to steer diets increased the prevalence of tetracycline-resistant *E*. *coli* (*P* < 0.02). Although the mechanism by which menthol exerts its effects remains unclear, results of our study suggest that menthol may have an impact on antimicrobial resistance in gut bacteria.

## Introduction

The rise of antimicrobial-resistant bacteria has been observed worldwide [1] and is a growing concern because of its potential to endanger the future of antimicrobial drug therapy [2]. Excessive use of therapeutic and non-therapeutic antimicrobials in human, animal health, and animal husbandry contributes to the emergence and dissemination of antimicrobial resistance (AMR; [3, 4]) in our environment (soil, water, etc…). Livestock, and more specifically ruminants’ gut flora, represents a large reservoir of antibiotic-resistant bacteria and resistance gene determinants, which can spread to the environment and to humans [5, 6]. The genes encoding for AMR, including multidrug resistance (MDR), are often carried on mobile genetic elements such as plasmids, transposons, and integrons [7, 8, 9], which facilitate horizontal transfer [10] from commensal to pathogenic bacteria and from livestock to human bacterial flora. *Escherichia coli*, a common gut bacterium in most mammal species and prevalent in feces of cattle [11], is often used to assess the impact of antimicrobial agents as it carries antibiotic resistance genes. Multiple antimicrobial resistance determinants have been found in *Escherichia coli* on the same plasmid, further facilitating their propagation and co-selection. For instance, the multidrug resistance plasmid IncA/C found in enteric bacteria, such as *Salmonella enterica* and *Escherichia coli*, often encodes for resistance to common antimicrobial agents such as tetracycline (*tetA*), chloramphenicol/florfenicol (*floR*), streptomycin/spectinomycin (*aadA2*), sulfonamides (*sul1* and *sul2*), and extended spectrum β-lactamases (*bla*_*CMY-2*_; [12]), and its spread to pathogenic bacteria may limit antibacterial means to fight infections caused by these bacteria. Therefore, compounds capable of limiting or preventing emergence of AMR and/or eliminating or inactivating mobile genetic elements may be of use in controlling antibiotic resistance dissemination, as well as MDR bacteria, and preserving antimicrobial efficacy.

Interest is considerable in using growth-promoting feed additives, such as probiotics, prebiotics, and plant-based compounds, as alternatives to antimicrobial agents to minimize the role of livestock as a reservoir of AMR bacteria [13, 14]. The impact of these non-antibiotic alternatives on prevalence and persistence of AMR bacteria in the gut has not yet been investigated in cattle *in vivo*. Menthol, a plant-based compound, is a monoterpene alcohol with known cooling and anesthetic properties, anti-pruritic activity, and antibacterial and antifungal activities [15]. Menthol has been shown to increase body weight gains in poultry [16–18]. In ruminants, inclusion of 3.3% menthol in a digestion trial [19] or 0.2% of peppermint oil in an *in vitro* ruminal fermentation assay [20] reduced protozoal, fungal, and bacterial populations in the rumen fluid. Direct addition of menthol to ruminal fluid in *in vitro* fermentation at concentrations greater than 0.1% was also shown to reduce total volatile fatty acid (VFA) concentrations [20]. However, nutrient digestibility tended to increase with 2.9% menthol in steers [19] and decrease with 5% menthol in lactating cows [21]. In addition to menthol’s inconsistent effect on animal growth performance, another interesting characteristic of menthol is its plasmid-curing activity. Schelz et al. [22] investigated the effects of peppermint oil and menthol *in vitro* on bacteria and their plasmids and demonstrated anti-plasmid activity similar to sodium dodecyl sulfate, which is a known plasmid-curing compound [23]. Because of menthol’s anti-plasmid activity, we postulated that inclusion of menthol in cattle diets could lead to reduction in the prevalence of MDR bacteria in the gut. Our objectives were to investigate the effects of menthol inclusion in the diet of feedlot cattle on fecal coliform populations and on AMR *Escherichia coli* in feces.

## Materials and Methods

Procedures for this study were approved by the Kansas State University Institutional Animal Care and Use Committee.

### Animals

Twenty-six Holstein steers (568.8 ± 55 kg body weight) were housed in individual pens within three barns containing 5, 5, and 3 steers representing each treatment. Barns had concrete-surfaced pens (1.5 m × 6 m), were covered with corrugated roofing and equipped with individual feed bunks. Water fountains were shared between adjacent pens. Two treatments, a control and a menthol group, were randomly assigned to steers and were equally represented in each of the three barns. Crushed menthol (99.7% purity, Prinova USA LLC, Carol Stream, IL) was included at 0.3% on a dry matter basis in a basal diet consisting of 50% steam flaked corn, 33% corn gluten feed, and 10% corn silage. Diets were manufactured daily to avoid excess volatilization of menthol. Steers received 300 mg of monensin (Elanco Animal Health, Greenfield, IN) and 90 mg of tylosin (Elanco Animal Health) per animal daily and were fed *ad libitum* with free access to water for 30 days.

### Sample collection and processing

Fecal samples were obtained from each animal from the rectum, before feeding, on days 0 (before inclusion of menthol), 16, 23, and 30. Samples were placed in plastic bags, kept on ice, and transported to the Kansas State University Preharvest Food Safety Laboratory. Fecal samples were stored at -80°C before analysis.

### Total coliform counts

Fecal samples obtained on days 0 and 30 were thawed and homogenized in a stomacher, and 1 g of each sample was suspended in 9 mL phosphate buffered saline (PBS) in a tube and vortexed. Fecal suspensions were allowed to settle, and a 50-μL of supernatant was spiral-plated, in duplicate, onto MacConkey agar using an Eddy Jet spiral plater (IUL instruments, Barcelona, Spain) and incubated overnight at 37°C. Lactose-fermenting colonies (coliform bacteria) were counted following spiral plating guidelines to determine coliform concentrations. If additional dilutions were needed, PBS was used to dilute the initial fecal suspension.

### *E*. *coli* isolation

A 100-μL volume of each fecal suspension from each of the sampling days (day 0, 16, 23, and 30) was spread-plated on MacConkey agar (BD Diagnostic Systems, Franklin Lakes, NJ) and incubated overnight at 37°C. A single lactose-fermenting colony was selected randomly from each plate and re-plated onto tryptic soy agar (TSA; Thermo Fisher Scientific, Lenexa, KS). After an overnight incubation at 37°C, colonies were tested for indole production by a spot indole test. Indole-positive colonies were stored on cryobeads (Cryocare; Key Scientific Products, Stamford, TX) at -80°C until further analysis.

### Minimum inhibitory concentration (MIC) determinations

Isolates from control and menthol groups were used to determine MIC for amoxicillin, ampicillin, azithromycin, cefoxitin, ceftiofur, ceftriaxone, chloramphenicol, ciprofloxacin, gentamicin, kanamycin, nalidixic acid, streptomycin, sulfisoxazole, tetracycline, and trimethropim/sulfamethoxazole (Gram-negative National Antimicrobial Resistance Monitoring System [NARMS] panel) using the broth microdilution method. Isolates stored in beads were grown on blood agar plates (Thermo Fisher Scientific Remel Products), and colonies were suspended in demineralized water (Trek Diagnostics Systems, Cleveland, OH) to obtain a suspension of 0.5 McFarland turbidity. A 50-μL aliquot of the suspension added to cation-adjusted Mueller-Hinton broth (Trek Diagnostics Systems) served as the inoculum. Tubes were vortexed and placed in the Sensititre automated inoculation delivery system (Trek Diagnostics Systems) to inoculate the Gram-negative NARMS panel plates (CMV2AGNF, Trek Diagnostics Systems). Plates were incubated for 18 h at 37°C and read manually using the Sensititre manual viewer (Sensitouch, Trek Diagnostics Systems). *E*. *coli* 25922 and *Staphylococcus aureus* 29213 strains (American Type Culture Collection, Manassas, VA) were used as quality control strains. Resistance or susceptibility of the isolates was determined based on CLSI guidelines [24].

### PCR detection of tetracycline resistance genes

Isolates that were phenotypically resistant to tetracycline were tested for *tet*A and *tet*B genes. DNA extraction was performed by suspending a single colony from a blood agar plate in 500 μL of deionized water in 1.5 mL microcentrifuge tube, boiling for 10 min at 100°C, and centrifuging at 10,000 x g for 5 min. A duplex PCR assay for *tet*A and *tet*B was performed as described by Harvey et al. [25] with *E*. *coli* ATCC 47042 (positive for *tet*B) and XL1-Blue *E*. *coli* strain (positive for *tet*A) as positive controls. The DNA in 96-well plates were amplified using an Eppendorf Mastercycler gradient thermal cycler (USA Scientific, Inc., Ocala, FL), and PCR products were then transferred to the Automated QIAxcel System (QIAgen, Valencia, CA). Microcapillary electrophoresis was performed using a QIAxcel DNA screening cartridge (QIAgen), a QX alignment marker (15bp/1 kb; QIAgen), and a 50 to 800 bp QX size marker (QIAgen). The electrophoresis was documented and analyzed for the presence of specific bands.

### Statistical analysis

Total coliform colony counts were log_10_ transformed and normality of the results was verified. Results were then analyzed with a generalized linear mixed model using the GLIMMIX procedure of SAS (9.2, Cary, NC). Treatment (control or menthol), sampling days (day 0, 16, 23, and 30), and their interaction were included in the model as fixed effects, and animal ID was used as the random effect. Unbiased least square means and standard errors were calculated using the LSMEANS statements of SAS and used to produce graphs.

Frequency analyses of resistant *E*. *coli* isolates to the multiple antibiotics tested were performed using the FREQ procedure of SAS with a chi-square test. Tetracycline resistance data were further analyzed with generalized linear mixed model using the GLIMMIX procedure of SAS with a binomial distribution, where treatment, sampling day, and their interaction were included as fixed effects, animal ID nested within treatment was used as the random effect, and tetracycline resistance status on day 0 was used as a covariate. Unbiased least square means and standard errors were calculated using the LSMEANS statements of SAS.

*E*. *coli* isolates were considered multidrug-resistant when resistant to 5 or more of the antibiotics tested. Multidrug-resistant bacteria, MDR phenotypes, *tet*A or *tet*B-positive genotypes, and the number of isolates resistant to tetracycline but not carrying *tet*A or *tet*B genes were analyzed with a chi-square test using the FREQ procedure of SAS. Further analysis of *tet*B genes was performed using a generalized linear mixed model in a GLIMMIX procedure with a binomial distribution where treatment and sampling day were included as fixed effects, animal ID nested within treatment was used as the random effect, and *tet*B resistance status on day 0 was used as a covariate. Unbiased least square means and standard errors were calculated using the LSMEANS statements of SAS. Low prevalence of *tet*B-positives genotypes precluded us from including in the model the interaction between sampling day and treatment.

Differences in least square means or frequencies were considered significant if the *P*-value was < 0.05.

## Results

Total colony counts of coliform bacteria in fecal samples of cattle fed diets with or without 0.3% menthol were 1.2 x 10^3^ and 6.0 x 10^2^ CFU/g on day 0 and 1.2 x 10^3^ and 3.6 x 10^3^ CFU/g on day 30, respectively. Total coliform counts were not affected by the day of sampling (*P* = 0.231) or the inclusion of menthol in the diet (*P* = 0.841), and there was no significant interaction between the day of sampling and the inclusion of menthol (*P* = 0.254). A total of 103 *E*. *coli* isolates (52 from the control and 51 from menthol groups) were tested to determine antimicrobial susceptibility patterns. The number and proportions of isolates resistant to antimicrobial agents, as determined by CLSI guidelines, are shown in Table 1. Frequency analyses showed that all isolates, regardless of sampling day or menthol treatment, were susceptible to azithromycin, ceftriaxone, ciprofloxacin, gentamicin, nalidixic acid, and sulfamethoxazole. In addition, overall *E*. *coli* isolates resistant to cefoxitin, amoxicillin, ceftiofur, or kanamycin were equal to or lower than 3.9%, and no differences in frequencies were observed between isolates originating from animals fed diets with and without menthol on any of the sampling days (*P* > 0.05). Chloramphenicol-, ampicillin-, and streptomycin-resistant isolates were found in 5.8, 5.8, and 7.7% of the control group samples and 3.9, 9.8, and 3.9% of the menthol group samples, respectively, but were not significantly different among treatments (*P* > 0.05). Of the *E*. *coli* isolates from steers fed the control and menthol diets, 94.4% and 88.2% were resistant to sulfisoxazole, respectively (*P* > 0.05). Tetracycline resistant isolates were found in 32.7%, respectively, of the control group and 56.9% of the menthol group (*P* = 0.014). Glimmix analysis for tetracycline resistance revealed no sampling day effect (*P* = 0.480), no menthol treatment effect (*P* = 0.093), but an interaction between sampling day and menthol treatment (*P* = 0.044).

**Table 1:** Antimicrobial susceptibilities of fecal *Escherichia coli* isolates from steers fed diets supplemented with or without 0.3% menthol.

	Number of resistant isolates/total isolates tested										
	Control				Menthol, 0.3%				Control	Menthol, 0.3%	
Antimicrobials	Day 0	Day 16	Day 23	Day 30	Day 0	Day 16	Day 23	Day 30	Total (%)		
**Amoxicillin**^**†**^	0/13	0/13	0/13	0/13	0/13	0/12	1/13	1/13	0/52 (0)		2/51 (3.9)
**Ampicillin**	1/13	1/13	1/13	0/13	1/13	0/12	2/13	2/13	3/52 (5.8)		5/51 (9.8)
**Azithromycin**	0/13	0/13	0/13	0/13	0/13	0/12	0/13	0/13	0/52 (0)		0/51 (0)
**Cefoxitin**	0/13	0/13	0/13	0/13	0/13	0/12	1/13	1/13	0/52 (0)		2/51 (3.9)
**Ceftiofur**	0/13	0/13	0/13	0/13	0/13	0/12	1/13	0/13	0/52 (0)		1/51 (2.0)
**Ceftriaxone**	0/13	0/13	0/13	0/13	0/13	0/12	0/13	0/13	0/52 (0)		0/51 (0)
**Chloramphenicol**	1/13	1/13	1/13	0/13	0/13	0/12	2/13	0/13	3/52 (5.8)		2/51 (3.9)
**Ciprofloxacin**	0/13	0/13	0/13	0/13	0/13	0/12	0/13	0/13	0/52 (0)		0/51 (0)
**Gentamicin**	0/13	0/13	0/13	0/13	0/13	0/12	0/13	0/13	0/52 (0)		0/51 (0)
**Kanamycin**	1/13	0/13	0/13	0/13	0/13	0/12	0/13	0/13	1/52 (1.9)		0/51 (0)
**Nalidixic acid**	0/13	0/13	0/13	0/13	0/13	0/12	0/13	0/13	0/52 (0)		0/51 (0)
**Streptomycin**	1/13	2/13	1/13	0/13	0/13	0/12	2/13	0/13	4/52 (7.7)		2/51 (3.9)
**Sulfamethoxazole**^**ф**^	0/13	0/13	0/13	0/13	0/13	0/12	0/13	0/13	0/52 (0)		0/51 (0)
**Sulfisoxazole**	11/13	11/13	12/13	13/13	13/13	9/12	12/13	11/13	47/52 (90.4)		45/51 (88.2)
**Tetracycline**	6/13	5/13	3/13	3/13^a^	8/13	3/12	8/13	10/13^b^	17/52 (32.7)		29/51 (56.9)

^ф^ with trimethoprim

† with clavulanic acid

^a, b^Values with different superscript letters are different; *P* < 0.02.

CLSI thresholds: Amoxicillin-clavulanic acid, R ≥ 32 μg/mL; ampicillin, R ≥ 32 μg/mL; azithromycin, R ≥ 16 μg/mL; ciprofloxacin, R ≥ 4 μg/mL; cefoxitin, R ≥ 32 μg/mL; ceftiofur, R ≥ 8 μg/mL; ceftriaxone, R ≥ 64 μg/mL; chloramphenicol, R ≥ 32 μg/mL; gentamicin, R ≥ 16 μg/mL; kanamycin, R ≥ 64 μg/mL; nalidixic acid, R ≥ 32 μg/mL; streptomycin, R ≥ 64 μg/mL; sulfamethoxazole, R ≥ 4 μg/mL; sulfisoxazole, R ≥ 256 μg/mL; tetracycline, R ≥ 16 μg/mL.

The proportion of isolates resistant to tetracycline was not different between treatments on day 16 (38.5% in the control group and 25% in the menthol group; *P* = 0.447), but tended to be greater in the menthol group on day 23 (23% in the control group and 61.5% in the menthol group; *P =* 0.084) and was greater on day 30 (*P =* 0.020; 23% in the control group and 76.9% in the menthol group).

Table 2 summarizes the prevalence of *tet*A and *tet*B genes in fecal *E*. *coli* phenotypically resistant to tetracycline in steers fed diets with or without menthol.

**Table 2:** Prevalence of *tet*A or *tet*B in fecal *Escherichia coli* phenotypically resistant to tetracycline in steers fed diets supplemented with or without 0.3% menthol.

Genes and treatment groups	Number of *tet*A- or *tet*B-positive/total number of isolate resistant to tetracycline (%)				
	Day 0	Day 16	Day 23	Day 30	Total
*tet*A					
Control	2/6 (33.3)	2/5 (40.0)	2/3 (66.7)	1/3 (33.3)	7/17 (41.2)
Menthol, 0.3%	0/8 (0.0)	2/3 (66.7)	3/8 (37.5)	1/10 (10)	6/29 (20.7)
*tet*B					
Control	2/6 (33.3)	1/5 (20.0)	0/3 (0.0)	1/3 (33.3)	4/17 (23.5)
Menthol, 0.3%	7/8 (87.5)	0/3 (0.0)	2/8 (25.0)	4/10 (40.0)	13/29 (44.8)

Overall, 41.2% of the isolates from the control group and 20.7% of the isolates from the menthol group carried *tet*A. Frequency analysis showed no difference in proportion of *tet*A positive isolates regardless of sampling day or menthol treatment (*P* = 0.592). Frequency analysis did, however, reveal an increase in proportion of *tet*B-positives isolates in the menthol treatment (44.8%) compared to the control (23.5%; *P* = 0.015). Isolates from animals fed menthol had greater prevalence of *tet*B than the control group on day 0 (87.5 and 33.3%, respectively; *P =* 0.039), but no significant difference among treatment were observed on day 16, 23, or 30 (*P >* 0.1). Those results were further investigated using the Glimmix analysis where *tet*B prevalence on day 0 was used as a covariate in the model. Results showed that number of *tet*B-positive isolates was not influenced by sampling day (*P* = 0.206) or by menthol treatment (*P* = 0.379).

All isolates carrying *tet*A or *tet*B were phenotypically resistant to tetracycline (MIC ≥ 16 μg/mL). Overall, 64.7% of the isolates resistant to tetracycline in the control group and 65.5% of the isolates resistant to tetracycline in the menthol group were found to carry *tet*A or *tet*B (P > 0.9). Conversely, 6 isolates from the control group (35.3%) and 10 from the high menthol group (34.5%) were resistant to tetracycline but did not carry *tet*A or *tet*B (Table 3; *P >* 0.9). On day 30, 50% of isolates in the menthol group and 33.3% of isolates in the control group were resistant to tetracycline and did not carry either *tet*A or *tet*B, but the difference was not a statistically significant (*P* = 0.850).

**Table 3:** Prevalence of tetracycline resistant isolates that do not carry *tet*A or *tet*B genes in fecal *Escherichia coli* in steers fed diets supplemented with or without 0.3% menthol.

	Isolates resistant to tetracycline that do not carry either *tet*A or *tet*B/ Number of tetracycline resistant isolates (%)				
Treatment	Day 0	Day 16	Day 23	Day 30	Total
Control	2/6 (33.3)	2/5 (40.0)	1/3 (33.3)	1/3 (33.3)	6/17 (35.3)
Menthol, 0.3%	1/8 (12.5)	1/3 (33.3)	3/8 (37.5)	5/10 (50.0)	10/29 (34.5)

Table 4 presents the percentages of MDR (≥ 5 antimicrobial agents) *E*. *coli* isolates in each treatment group. Frequency analysis showed that overall prevalence of MDR isolates was not different in the control group (3.8%) or in the 0.3% menthol (5.9%; *P* > 0.631). Additionally, there was no difference in MDR frequency between treatments on days 0, 16, 23, and 30 (*P* > 0.3).

**Table 4:** Multidrug resistance (MDR; ≥ 5 antimicrobials) prevalence (%) in fecal *Escherichia coli* from steers fed diets supplemented with or without 0.3% menthol.

	Number of MDR isolates/total isolates tested (%)				
Treatment	Day 0	Day 16	Day 23	Day 30	Total
Control	0/13 (0)	1/13 (7.7)	1/13 (7.7)	0/13 (0)	2/52 (3.8)
Menthol, 0.3%	0/13 (0)	0/12 (0)	2/13 (15.4)	1/13 (7.7)	3/51 (5.9)

Table 5 illustrates the various antibiotic resistance phenotypes among the *E*. *coli* isolates tested. Only 4.8% of the total isolates were pan-susceptible. Of the isolates tested, 50.5% were resistant to sulfisoxazole only, and 30.1% were resistant to both sulfisoxazole and tetracycline. Only one isolate from the menthol group was found to be resistant to eight antibiotics.

**Table 5:** Prevalence of antimicrobial-resistant fecal *Escherichia coli* isolates from cattle fed diets with or without 0.3% menthol.

Phenotypes	Control	Menthol, 0.3%	Total (%)
_AMP_CHL_STR_FIS_TET	2	1	3 (2.9)
_AMP_FIS_TET	0	2	2 (1.9)
_AMP_STR_FIS_TET	1	0	1 (1.0)
_AUG_AMP_FOX_FIS_TET	0	1	1 (1.0)
_AUG_AMP_FOX_XNL_CHL_STR_FIS_TET	0	1	1 (1.0)
_CHL_KAN_FIS_TET	1	0	1 (1.0)
_FIS	32	20	52 (50.5)
_FIS_TET	11	20	31 (30.1)
_TET	1	4	5 (4.8)
Pan susceptible	3	2	5 (4.8)

Amoxicillin-clavulanic acid, AUG; ampicillin, AMP; azithromycin, AZI; ciprofloxacin, CIP; cefoxitin, FOX; ceftiofur, XNL; ceftriaxone, AXO; chloramphenicol, CHL; gentamicin, GEN; kanamycin, KAN; nalidixic acid, NAL; streptomycin, STR; sulfamethoxazole, SXT; sulfisoxazole, FIS; tetracycline, TET.

## Discussion

Based on the known antimicrobial activity of menthol against *E*. *coli*, we hypothesized that menthol would impact total coliform counts in cattle fecal samples. Menthol metabolism in the rumen is poorly understood, leading us to investigate the impact of 0.3% dietary menthol on commensal coliform populations in feedlot cattle. Menthol concentration in ruminal contents was not measured in this experiment, but can be roughly estimated at 2.7 mM considering a 50 L ruminal volume and 7 kg daily dry matter intake [26]. Previous studies have demonstrated inhibitory effects with concentrations of 75 mM for *E*. *coli* O157:H7 [27] and as low as 16 mM with *E*. *coli* ATCC15221 [28]. The lack of difference between total fecal coliform counts in the control group and menthol-supplemented groups suggests either that the level of menthol reaching the hindgut was insufficient to inhibit the organism or that bacteria were able to adapt to menthol presence within the gut. Landau and Shapira [27] recently showed enterohemorrhagic *E*. *coli* (EHEC) to have the ability to adapt to increasing levels of subinhibitory concentration of menthol, and a similar adaptation processes could be anticipated for other *E*. *coli*.

Our main objective was to investigate if menthol inclusion in feedlot diets would affect *E*. *coli* resistance to antibiotics and the prevalence of MDR organisms. Although total *E*. *coli* populations and MDR *E*. *coli* were not affected by 30 days of menthol supplementation, we analyzed individual minimum inhibitory concentration (MIC) of fecal *E*. *coli* isolates from the control group and the group receiving 0.3% of menthol daily after 0, 16, 23, and 30 days of exposure to treatments. Results of MIC evaluations revealed that all 103 isolates were susceptible to azithromycin, ceftriaxone, ciprofloxacin, gentamicin, and nalidixic acid, sulfamethoxazole, and only a small percentage of isolates were resistant to amoxicillin, cefoxitin, ceftiofur, and kanamycin, regardless of sampling day and of treatment received by the animals. Similar observations were made by Mirzaagha et al. [29], who found all 531 *E*. *coli* isolates collected from feedlot cattle fed diets with and without chlortetracycline and/or sulfamethazine susceptible to ceftriaxone, cefoxitin, gentamicin, and nalidixic acid. Gow et al. [30] also failed to detect any fecal *E*. *coli* resistance to ceftriaxone, ciprofloxacin, or nalidixic acid among the 207 isolates collected from cow-calf herds in western Canada and observed that only 1% were resistant to gentamicin, 1.5% to ceftiofur, and 4.8% to amoxicillin and cefoxitin. They did, however, observe greater resistance rates for kanamycin (15%) and sulfamethoxazole (55.1%) compared with our study. Chloramphenicol resistance, like previous antibiotics, was not affected by menthol treatment in our experiment. The presence of resistant isolates (4.8% overall) was somewhat surprising, as chloramphenicol use in animal production systems was banned more than 30 years ago [31]. Our observations are, however, not unusual; others have reported even higher prevalence (14.5 to 31%) in commensal *E*. *coli* from cattle [30, 32, 33]. Persistence of chloramphenicol resistance in the environment is thought to be due to the use of closely related antibiotics, such as florfenicol, or to a co-selection phenomenon [34]. Unfortunately, low prevalence of chloramphenicol-resistant isolates in this study did not allow us to reveal a resistance pattern associated with the presence of chloramphenicol resistance. Like chloramphenicol, the prevalence of isolates resistant to streptomycin was not affected by sampling day or menthol treatment. Overall, 5.8% of *E*. *coli* isolates tested in this experiment were resistant to streptomycin. Gow et al. [30] reported 41.6% *E*. *coli* resistant isolates from cow-calf herds, and Ma et al. [32] found that 89.1% of *E*. *coli* isolates from dairy cows were resistant. Differences in animal production system practices could explain the lower prevalence observed in our study, as animal exposures to antimicrobials are likely to be different. Resistance to sulfisoxazole was found in 89.3% of the *E*. *coli* isolates tested in our study and was not influenced by menthol inclusion in the diet. A large-scale study conducted in a feedlot in Texas also reported high resistance rate, with 65% of the tested 7,097 *E*. *coli* isolates resistant to sulfisoxazole. Prevalence in the Texas study was not influenced by the type of growth promotants received by the animals [35], which further underscores the widespread nature of sulfisoxazole resistance determinants in commensal bacteria. Overall ampicillin resistance, 7.8%, was not affected by the inclusion of menthol in the diets. This prevalence was lower than previously observed prevalence in *E*. *coli* from cattle, which has ranged from 18 to 48% [30, 33, 36]. Menthol supplementation did, however, have a significant effect on tetracycline resistance. After 30 days of menthol treatment, 76.9% of the isolates from the menthol group tested resistant to tetracycline compared with only 23.1% in the control group. Moreover, *E*. *coli* isolates resistant to tetracycline within the menthol group increased from day 16 to day 23 (25% and 61.5%, respectively) and from day 23 to day 30 (61.5% and 76.9%, respectively), while control group remained fairly constant (38.5% on day 16 and 23.1% on day 23 and 30). These observations underlined an effect of menthol on tetracycline resistance phenotypes, which compelled us to further investigate tetracycline genotype profiles of these isolates.

There are 40 known *tet* resistance determinants most of which are found on mobile genetic elements that encode for efflux pump [37]. *Tet*A and *tet*B are the most prevalent genes in tetracycline resistant *E*. *coli* [38], which is why we chose to focus on these two. *Tet*A and *tet*B encode for an efflux pump in the lipid bilayer of the bacteria, which removes the tetracycline/cation complex from the cell by exchanging a proton [39]. *Tet*B is usually more predominant than *tet*A and is linked to higher MIC [40]. Out of the 103 isolates investigated in our experiment, 16.5% were found to carry *tet*B and 13.6% to carry *tet*A. Moreover, no isolates were found to carry both determinants, which corroborates previous findings [40, 41]. *Tet*A and *tet*B are believed to be located on plasmids, though from different incompatibility groups [41]. This potentially explains why they rarely are detected together in bacteria. The absence of an effect of menthol inclusion on *tet*A seems to exclude the implication of *tet*A in the difference observed in tetracycline phenotypes in *E*. *coli* isolates from steers fed diets with and without menthol. In addition, the greater prevalence of *tet*B in the menthol group compared to the control on day 0 and the absence of an effect of menthol inclusion on *tet*B prevalence also exclude the implication of *tet*B in the difference observed in tetracycline phenotypes. Despite the greater prevalence of tetracycline resistance in the menthol group, frequency of *E*. *coli* isolates that were phenotypically resistant to tetracycline, though not carrying either *tet*A or *tet*B, was not significantly affected by the menthol treatment. Based on our results, the observed increase in tetracycline resistance also was not explained by the presence of other *tet* resistance determinants in *E*. *coli* isolates from steers fed 0.3% menthol; however, the small sample size of phenotypically tetracycline resistant isolates (46 isolates) may have limited our ability to detect statistically meaningful differences.

In conclusion, menthol supplementation of feedlot diets at a 0.3% rate for 30 days did not alter the total coliform population in fecal samples and did not affect prevalence of resistance to azithromycin, ceftriaxone, ciprofloxacin, gentamicin, nalidixic acid, cefoxitin, amoxicillin, ceftiofur, sulfamethoxazole, kanamycin, streptomycin, sulfisoxazole, chloramphenicol, and ampicillin. Menthol supplementation did, however, increase the prevalence of tetracycline-resistant *E*. *coli* isolates, but did not affect *tet*A and *tet*B gene-positive *E*. *coli* or the number of MDR bacteria. The underlying mechanism associated with this increase in tetracycline resistance is unknown; nevertheless, this study demonstrates a possible effect of menthol on bacterial antimicrobial resistance. If antibiotic substitutes, such as menthol, do indeed influence prevalence of AMR in gut bacteria, this raises questions concerning the appropriateness of these compounds as alternatives to traditional antibiotics.

## Supporting Information

S1 Dataset Antimicrobial susceptibilities, phenotypes, MDR of fecal *Escherichia coli* isolates from steers fed diets supplemented with or without 0.3% menthol. (XLS)
